# Which indicators of early cancer diagnosis from population-based data sources are associated with short-term mortality and survival?

**DOI:** 10.1016/j.canep.2018.07.010

**Published:** 2018-10

**Authors:** Patrick Muller, Sarah Walters, Michel P. Coleman, Laura Woods

**Affiliations:** Cancer Survival Group, Department of Non-Communicable Disease Epidemiology, London School of Hygiene and Tropical Medicine, Keppel Street, London WC1E 7HT, UK

**Keywords:** Cancer mortality, Surveillance, Cancer survival, Diagnostic interval, Early detection, Early diagnosis, Emergency presentation, Population-based, Routine data, Stage at diagnosis

## Abstract

**Background:**

A key component of recent English cancer policy is the monitoring of trends in early diagnosis of cancer. Early diagnosis can be defined by the disease stage at diagnosis or by other indicators derived from electronic health records. We evaluate the association between different early diagnosis indicators and survival, and discuss the implementation of the indicators in surveillance of early diagnosis.

**Methods:**

We searched the PubMed database and grey literature to identify early diagnosis indicators and evaluate their association with survival. We analysed cancer registrations for 355,502 cancer patients diagnosed in England during the period 2009–2013, and quantified the association between each early diagnosis indicator and 30-day mortality and five-year net survival.

**Results:**

Each incremental difference in stage (I–IV) predicts lower 5-year survival, so prognostic information is lost in comparisons which use binary stage indicators. Patients without a recorded stage have high risk of death shortly following diagnosis and lower 5-year survival. Emergency presentation is independently associated with lower five-year survival. Shorter intervals between first symptoms and diagnosis are not consistently associated with improved survival, potentially due to confounding from tumour characteristics.

**Interpretation:**

Contrary to current practice, we recommend that all the stage information should be used in surveillance. Patients missing stage should also be included to minimise bias. Combined data on stage and emergency presentation could be used to create summary prognostic measures. More work is needed to create statistics based on the diagnostic interval that will be useful for surveillance.

## Highlights

• Each difference in tumour stage (I–IV) predicts lower five-year survival, so prognostic information is lost in binary indicators.
• Emergency presentation is associated with lower survival, independently of stage.
• A high proportion of patients whose stage is not recorded die immediately after diagnosis.
• Interval from first symptoms to diagnosis is not consistently associated with survival.

## 1 Introduction

Increasing early-stage diagnosis is a common component of regional and national strategies to reduce the burden of cancer [[1], [2], [3], [4], [5]]. ‘Early diagnosis’ is often used as a shorthand for ‘early-stage diagnosis’, which has historically been the outcome in early diagnosis studies. However, alternative indicators based on electronic health records are increasingly being used in early diagnosis studies. Some of these indicators relate to the promptness of diagnosis following clinical presentation, or the health services patients accessed first [6].

In England, cancer surveillance statistics are published on Public Health England’s ‘CancerData’ dashboard for each of the 209 local healthcare commissioning bodies (Clinical Commissioning Groups – CCGs) [7]. These include the percentage of patients diagnosed with localised tumours (TNM Stage I/II), the percentage diagnosed following emergency admission or referral, and statistics on adherence to targets for patient waiting times.

Surveillance in England was only initiated in 2016, and optimal implementation of the different possible indicators in surveillance has not been extensively researched. Information on the association between each indicator and short-term mortality and survival will help analysts interpret the indicators, and identify those which are timely measures of progress in raising survival from cancer.

In this study, we report a systematic literature review and data analysis. Our aim is to identify early diagnosis indicators and evaluate the association between each indicator and short-term mortality and survival. We then discuss the implications of our findings for surveillance.

## 2 Methods

We conducted a systematic literature search to identify indicators of early cancer diagnosis in population-based data sources and evaluate their association with short-term mortality and survival. The association between each indicator and (i) risk of death within 30 days following diagnosis (“30-day mortality”) (ii) net survival at one and five years was then analysed for patients diagnosed in 2009–2013 in England with either colorectal cancer, non-small cell lung cancer (NSCLC) or ovarian cancer, malignancies frequently diagnosed at advanced stage [8].

### 2.1 Literature review

#### 2.1.1 PubMed search

Journal articles published between August 2007 and August 2017 were examined [9]. Articles that contained each of three keyword elements (cancer, early diagnosis, population-based) in the title or the abstract were retained (Appendix A in Supplementary data).

#### 2.1.2 Google.com search

Google was searched using the same keywords on 25 August 2017. The first 20 hyperlinks returned were exhaustively searched for relevant journal articles or reports, and any found were retained.

#### 2.1.3 Document selection strategy

The abstract of each retained article was read. If it reported new statistics or methods for generating statistics based on an indicator of early diagnosis from population-based data sources (inclusion criterion 1, Appendix A in Supplementary data), the article’s full text was read.

The summary, introduction and conclusion of retained reports were read. Those meeting the above inclusion criterion were identified. Relevant portions of the full text of these reports were then read.

Details regarding the early diagnosis indicators and their association with any measure related to survival (complications, mortality, survival, life expectancy, or cure (inclusion criterion 2, Appendix A in Supplementary data)) were extracted.

### 2.2 Data analysis

#### 2.2.1 Patient cohort

Cancer registrations were obtained from the Office for National Statistics (ONS) for adults aged 15–99 years, diagnosed with colorectal cancer, NSCLC or ovarian cancer in England in 2006–2013 (ICD-10 codes C18-20, C21.8, C33-34 and C56-C57.7 [10]). Follow-up was complete up to 31 December 2014. Registrations were linked to datasets from the National Bowel and Lung Cancer Audits, and to the Routes to Diagnosis dataset [11] using the patient’s NHS number and postcode. These datasets were used to complete information on stage at diagnosis [12].

#### 2.2.2 Data analysis

Thirty-day mortality and one- and five-year net survival were estimated by agegroup (15–59, 60–79, 80–99 years). Net survival estimates were obtained using Pohar Perme’s unbiased estimator [13] and the period approach applied to follow-up data during 2009–2013 for patients diagnosed during 2006–2013 [14] (details in Appendix B in Supplementary data).

To avoid unstable sub-group estimates, one-year survival was only estimated if, in the period 2009–2013, at least 25 patients were diagnosed and five deaths occurred within the first year after diagnosis. Five-year survival was estimated if at least 15 patients were alive at one year after diagnosis and five deaths occurred in the second to fifth years. Missing data was either included in a separate category, or excluded (complete-case analysis).

## 3 Results

### 3.1 Literature search

**Fig. 1:**
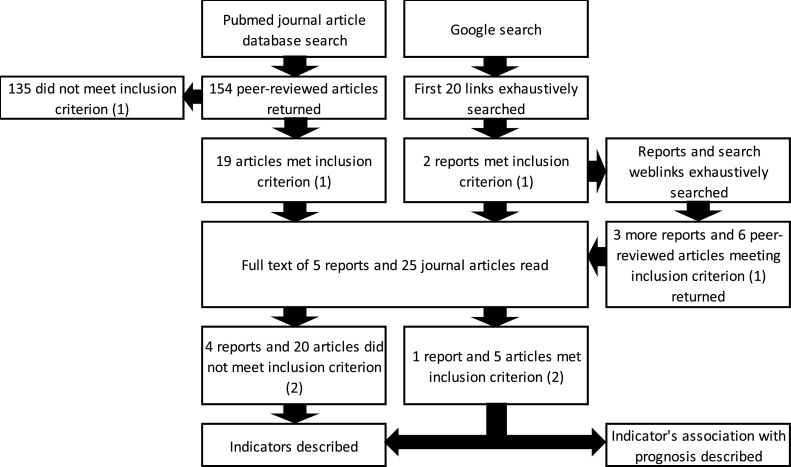
Search strategy with number of reports and journal articles which reported new early diagnosis statistics or methods for generating these (inclusion criterion (1)), and number reporting the association between an indicator and a measure related to survival (inclusion criterion (2)).

The PubMed search returned 154 articles (Fig. 1), 19 of which presented new statistics or methods for generating statistics for an early diagnosis indicator. The Google search returned five reports and six articles also meeting that criterion.

Three early diagnosis indicators were identified in these 30 documents: stage at diagnosis (21 documents), emergency admission or emergency presentation (five), and interval from first symptom to diagnosis (eight).

Four documents contained information on survival and two on mortality.

#### 3.1.1 Indicator 1: Stage at diagnosis

##### 3.1.1.1 Definition and description

Stage was the sole indicator used in 18 documents (Table 1), and was one of several indicators in a further three. Typically the TNM classification system was used, directly or using ordinal stage (I–IV). Dukes’ stage for colorectal cancer [16] and tumour thickness for melanoma [23] were also used. Stage was frequently dichotomised into ‘early’ (localised, stage I or II, non-metastatic) vs. ‘late’ (advanced, stage III or IV, metastatic) [16,22]. The *CancerData* dashboard uses a binary indicator for whether the patient has a record of stage I/II disease, and presents this as a percentage of all patients (including those without a recorded stage) [37]. Other studies imputed stage information [15,19], or analysed missing stage as a separate group [35]. Average stage at diagnosis varied by tumour site [38], histological type for ovarian cancer [20], and by subsite for colorectal cancer [33].

**Table 1:** Cancers investigated, early diagnosis indicators and survival measures used in the documents read in full.

Reference		Early diagnosis indicator	Survival measure	Document type	Cancer(s)	Study population
Ahrensberg et al. [17]	∼	Interval from first presentation to diagnosis	–	Journal article	Childhood cancers/benign tumours of the Central nervous system (CNS)	Population-based analysis of Danish children diagnosed in 2007–2010
Aitken et al. [23]	^	Tumour thickness (one component of TNM stage)	–	Journal article	Melanoma	Population-based analysis of Australian people aged 20–75 diagnosed in 2000–2003
Castanon et al. [24]	^	Stage†	–	Journal article	Cervix	Population-based analysis of English and Welsh women aged 30–69 diagnosed in 1990–2014
Chorley et al. [26]	^	Stage	–	Journal article	Cervix, breast, colorectal	Survey of English adults aged 50–70 conducted in 2015
Ciocan et al. [25]	^	Stage	–	Journal article	Melanoma	Population-based analysis of French people diagnosed with melanoma in 2004–2008
Durbec et al. [27]	^	Tumour thickness (one component of TNM stage)	–	Journal article	Melanoma	Population-based analysis of French people diagnosed in 2004
Elliss-Brookes et al. [45]	¥	Emergency presentation	Relative survival from diagnosis	Journal article	15 common cancers	Population-based analysis of English people diagnosed in 2006–2008
Eskesen et al. [28]	^	Stage	–	Journal article	Liver	Population-based analysis of Norwegian people diagnosed in 2000–2009
Ess et al. [29]	^	Stage	–	Journal article	Breast	Representative sample study of Swiss women diagnosed in 2003–2005
Greenlee et al. [30]	^	Stage (Local vs distant)	–	Journal article	Larynx, oral cavity, melanoma, breast, prostate, corpus uteri, cervix, bladder, colorectum, esophagus, stomach, kidney	Population-based analysis of people in the USA diagnosed in 1997–2000
Gupta et al. [18]	∼	Interval from first presentation to diagnosis	Event-free survival from diagnosis	Journal article	Acute lymphoblastic leukaemia	Population-based analysis of Canadian children diagnosed in 1995–2011
Hamilton et al. [43]	∼	Interval from first symptoms to diagnosis	Mortality amongst symptomatic patients	Journal article	21 common cancers	Qualitative survey of 22 UK and Danish experts conducted in 2014
Hreinsson et al. [31]	^	Stage (I-IV, non-metastatic vs metastatic)	–	Journal article	Colorectal	Population-based analysis of Irish people diagnosed 2008–2011
Independent Cancer Taskforce [38]	^ ¥ ∼	Stage, emergency presentation, interval from first recognition of symptoms to diagnosis	–	Report	All cancers	None, specification of an early diagnosis statistic
Laudicella et al. [19]	^	Stage I/II vs III/IV	–	Journal article	Colorectal, prostate, lung, breast	Population-based analysis of English women diagnosed in 2001–2010
Li et al. [15]	^	Stage	Net cancer survival from diagnosis	Journal article	Breast	Population-based analysis of English women diagnosed in 2000–2007
Lurie et al. [32]	^	Stage	–	Journal article	Ovarian	Population-based analysis of women in the USA diagnosed in 1993–1997
Lurie et al. [20]	^	Stage	–	Journal article	Ovarian	Population-based analysis of women diagnosed in the USA in 1993–2008
Lyratzopoulos et al. [49]	∼	Interval from first recognition of symptoms to referral, broken into patient and primary care intervals	–	Journal article	28 adult cancers	Population-based study of English patients presenting in primary care in 2009–2010
Murage et al. [16]	^ ¥	Stage (Duke's AB vs CD), Emergency admission		Journal article	Colorectal	Population-based analysis of Scottish patients diagnosed in 1998–2011
National Cancer Intelligence Network [35]	^	Stage	Net cancer survival from diagnosis	Report	Colorectal	Population-based study of adults diagnosed in England in 1996–2002
Public Health England [36]	¥	Emergency presentation	–	Report	All invasive malignancies	None, specification of an early diagnosis statistic
Public Health England [37]	^	Stage (I, II vs III, IV, missing)	–	Report	Breast, prostate, colorectal, lung, baldder, kidney, ovary, uterus, non-Hodgkin lymphomas, melanoma	None, specification of an early diagnosis statistic
Rubin et al. [41]	¥	Emergency presentation	–	Journal article	All cancers	Mixed-methods (interviews and data analysis) study of the English GP practices in the period 2009–2013
Sala et al. [21]	^	Stage	–	Journal article	Breast	Population-based analysis of Spanish women aged 50-69 who attended screening in 1995–2010
Sankaranarayanan et al. [33]	^	Stage (localised, in-situ vs regional, distant)	–	Journal article	Colorectal	Population-based analysis of people in the USA diagnosed 1998–2003
Torring et al. [42]	∼	Interval from first presentation to diagnosis	Mortality risk within 5 years of diagnosis	Journal article	Colorectal, lung, melanoma, breast, prostate	Population-based analysis of Danish adults diagnosed in 2004–2005
Tracey et al. [22]	^	Stage (localised vs advanced)	–	Journal article	[All cancers]	Population-based analysis for Australian people diagnosed in 1980–2008
Walter et al. [40]	∼	Interval from first recognition of symptoms to diagnosis	–	Journal article	Lung	Population-based study of English people referred to secondary care in 2010–2012
World Health Organisation [34]	^ ∼	Stage, interval from first recognition of symptoms to diagnosis	–	Report	All cancers	None, specification of an early diagnosis statistic

^†^“Stage” = “Stage of disease at diagnosis”.

^^^Stage.

^¥^Emergency presentation.

^∼^Interval from firsy symptoms to diagnosis.

##### 3.1.1.2 Association with patient survival

Early-stage disease was associated with higher survival (net, relative or unadjusted). Women diagnosed with breast cancer during 2000–2007 in northeast England had one-year net survival over 90% at stages I-III, but only 50% at stage IV [15]. Five-year survival was high for women diagnosed at stage I/II, substantially lower for stage III and lower still for stage IV. There was a similar pattern for patients diagnosed in England during 1996–2002 with colorectal cancer: five-year survival was 93.2% for the patients diagnosed with Dukes’ A, 47.7% for Dukes’ C, and 6.6% for stage D [35].

#### 3.1.2 Indicator 2: Emergency admission or presentation

##### 3.1.2.1 Definition and description

Emergency admission or presentation was mentioned in five documents (Table 1). This is defined as an admission coded as ‘emergency’ and/or ‘accident & emergency’ [16], or a route to diagnosis via the Accident & Emergency department or via an emergency referral or transfer [11].

##### 3.1.2.2 Association with short-term mortality and survival

Elliss-Brookes et al. [11] found an association between emergency presentation and 1-year relative survival, noting “the substantially lower relative survival in emergency compared to non-emergency routes indicates that this distinction is of high clinical significance”.

#### 3.1.3 Indicator 3: Interval from first symptoms to diagnosis

##### 3.1.3.1 Definition and description

Time from first cancer-relevant symptom to referral or diagnosis was used in eight documents (Table 1).

The interval start was the time the patient first noticed symptoms [34,39,40] or time of presentation with symptoms to the GP [17,18,42]. The relevance of a symptom to cancer was decided by the GP [42], specialist clinician review [18], or by reference to external standards [40].

The end-point was cancer diagnosis or referral to secondary care. One study defined two intervals: the patient interval (time from symptom onset to first clinical presentation), and the primary care interval (time from first clinical presentation to specialist referral) [39].

##### 3.1.3.2 Association with short-term mortality and survival

The association between the intervals and survival varied by cancer. One study of childhood acute lymphoblastic leukaemia (ALL) found a prolonged interval from presentation to diagnosis was associated with longer event-free survival, although this was attributed to confounding from disease biology [18]. Another study found a ‘U-shaped’ curve between interval and odds of death within five years for five common cancers, with higher odds for patients with the shortest and longest intervals [42]. The high odds of death amongst patients with short intervals was attributed to confounding, arising because of GPs expediting diagnosis for patients with high-risk symptoms.

In one survey of expert judgement for 21 common cancers [43,44] there was consensus that expedited diagnosis brings mortality reductions for 11 cancers. They were undecided for seven cancers, and disagreed that expedited diagnosis conferred any mortality benefit for three.

### 3.2 Data analysis

We analysed the association between stage at diagnosis and emergency presentation with 30-day mortality and survival for 160,617 colorectal, 170,425 non-small cell lung (NSCLC), and 24,450 ovarian cancer patients (Table 2). Data was not available to examine the interval from first symptoms to diagnosis.

**Table 2:** Number of patients (%), and risk of death within 30 days following diagnosis (Mortality) by age group, route, cancer, and stage at diagnosis, England 2009–2013.

	All ages						Ages 15–59						Ages 60–79						Ages 80–99					
	All routes			Emergency presentation only			All routes			Emergency presentation only			All routes			Emergency presentation only			All routes			Emergency presentation only		
	N	(%)	*Mortalty %*	N	(%)	*Mortalty %*	N	(%)	*Mortalty %*	N	(%)	*Mortalty %*	N	(%)	*Mortalty %*	N	(%)	*Mortalty %*	N	(%)	*Mortalty %*	N	(%)	*Mortalty %*
Colorectal cancer																								
I	17,180	(10.9)	*0.4*	1,142	(3.1)	*3.4*	2,752	(10.7)	*0.1*	314	(5.2)	*0.6*	11,173	(12.5)	*0.2*	420	(2.6)	*1.2*	3,255	(7.6)	*1.6*	408	(2.7)	*7.8*
II	27,579	(17.5)	*2.0*	5,158	(13.8)	*7.4*	4,056	(15.7)	*0.2*	843	(13.8)	*0.8*	16,056	(18.0)	*1.3*	2,444	(15.0)	*5.7*	7,467	(17.5)	*4.5*	1,871	(12.6)	*12.5*
III	29,680	(18.8)	*1.8*	5,788	(15.5)	*6.3*	5,528	(21.4)	*0.2*	1,064	(17.5)	*0.8*	17,479	(19.6)	*1.3*	2,833	(17.3)	*5.2*	6,673	(15.7)	*4.4*	1,891	(12.7)	*10.9*
IV	33,215	(21.1)	*11.2*	11,059	(29.7)	*23.9*	6,300	(24.4)	*4.9*	1,965	(32.2)	*10.1*	18,283	(20.5)	*9.4*	5,329	(32.6)	*21.5*	8,632	(20.3)	*19.8*	3,765	(25.4)	*34.4*
Missing	50,125	(31.8)	*10.6*	14,138	(37.9)	*26.4*	7,151	(27.7)	*3.1*	1,909	(31.3)	*7.1*	26,380	(29.5)	*7.1*	5,312	(32.5)	*22.4*	16,594	(38.9)	*19.4*	6,917	(46.6)	*34.8*
Total	157,779	(100.0)	*6.5*	37,285	(100.0)	*19.2*	25,787	(100.0)	*2.1*	6,095	(100.0)	*5.8*	89,371	(100.0)	*4.5*	16,338	(100.0)	*16.1*	42,621	(100.0)	*13.2*	14,852	(100.0)	*28.1*
Non-small cell lung cancer																								
I	19,018	(11.4)	*2.3*	3,440	(5.7)	*7.9*	2,053	(9.6)	*0.4*	233	(3.6)	*1.3*	12,303	(12.1)	*1.9*	1,769	(5.4)	*6.6*	4,662	(10.7)	*4.3*	1,438	(6.7)	*10.5*
II	10,921	(6.6)	*3.6*	2,127	(3.5)	*11.2*	1,268	(5.9)	*1.0*	165	(2.5)	*4.2*	6,968	(6.9)	*3.1*	1,095	(3.4)	*11.1*	2,685	(6.1)	*6.3*	867	(4.0)	*12.7*
III	34,151	(20.5)	*7.8*	8,316	(13.7)	*20.4*	4,466	(20.9)	*4.1*	818	(12.6)	*13.2*	21,795	(21.5)	*6.6*	4,587	(14.1)	*18.4*	7,890	(18.0)	*13.1*	2,911	(13.5)	*25.8*
IV	75,506	(45.4)	*22.8*	33,168	(54.7)	*36.8*	10,708	(50.1)	*14.5*	4,188	(64.5)	*24.7*	46,260	(45.7)	*21.5*	18,755	(57.6)	*36.0*	18,538	(42.4)	*31.0*	10,225	(47.3)	*43.4*
Missing	26,817	(16.1)	*37.2*	13,581	(22.4)	*51.0*	2,865	(13.4)	*22.3*	1,086	(16.7)	*39.7*	14,004	(13.8)	*35.2*	6,327	(19.4)	*51.3*	9,948	(22.8)	*44.4*	6,168	(28.5)	*52.7*
Total	166,413	(100.0)	*18.5*	60,632	(100.0)	*35.2*	21,360	(100.0)	*11.2*	6,490	(100.0)	*24.4*	101,330	(100.0)	*16.5*	32,533	(100.0)	*34.0*	43,723	(100.0)	*26.5*	21,609	(100.0)	*40.3*
Ovarian cancer																								
I	3,989	(16.7)	*0.6*	472	(6.3)	*2.3*	1,983	(27.3)	*0.1*	282	(16.5)	*0.7*	1,688	(13.7)	*0.4*	147	(4.0)	*2.0*	318	(7.3)	*4.4*	43	(2.0)	*14.0*
II	1173	(4.9)	*1.9*	147	(2.0)	*8.2*	449	(6.2)	*0.7*	56	(3.3)	*3.6*	591	(4.8)	*1.4*	65	(1.8)	*6.2*	133	(3.0)	*8.3*	26	(1.2)	*23.1*
III	5,745	(24.0)	*4.0*	1,555	(20.7)	*10.0*	1,672	(23.0)	*1.3*	423	(24.7)	*3.3*	3,329	(27.1)	*3.4*	859	(23.6)	*8.5*	744	(17.1)	*12.5*	273	(12.7)	*24.9*
IV	4,288	(17.9)	*12.0*	1,858	(24.8)	*20.5*	956	(13.2)	*4.5*	360	(21.0)	*8.9*	2,432	(19.8)	*9.4*	992	(27.3)	*16.4*	900	(20.6)	*26.8*	506	(23.5)	*36.8*
Missing	8,712	(36.4)	*16.9*	3,471	(46.3)	*30.7*	2,206	(30.4)	*5.3*	592	(34.6)	*12.5*	4,240	(34.5)	*13.8*	1,572	(43.2)	*26.1*	2,266	(52.0)	*34.0*	1,307	(60.6)	*44.3*
Total	23,907	(100.0)	*9.5*	7,503	(100.0)	*21.6*	7,266	(100.0)	*2.6*	1,713	(100.0)	*7.2*	12,280	(100.0)	*7.7*	3,635	(100.0)	*18.0*	4,361	(100.0)	*25.9*	2,155	(100.0)	*39.2*

#### 3.2.1 Stage at diagnosis: association with 30-day mortality and net survival

Stage was missing for a large proportion of patients in the linked datasets we analysed (16.1–36.4% of patients for the three cancers). Colorectal and NSCLC patients aged 60–79 were most likely to be diagnosed at stages I or II (43% and 22% respectively), but there were not substantial differences between age groups (Table 2). By contrast, ovarian cancer patients aged 15–59 were considerably more likely to be diagnosed at stage I/II than those aged 80–99 (48% vs 22%).

Risk of 30-day mortality was higher at higher stages of disease, for all cancers and age groups, with the exception of colorectal cancer where mortality risk plateaued at stages II-III (Table 2). Thirty-day mortality was considerably higher for stage IV patients than stage III patients: six-times higher for colorectal cancer and three-times higher for NSCLC and ovarian cancer patients. NSCLC and ovarian cancer patients with missing stage had even higher mortality than stage IV patients (37.2% vs. 22.8% for NSCLC; 16.9% vs.12% for ovarian cancer) whereas mortality for colorectal cancer patients with missing stage was between that of patients diagnosed at stages III and IV.

**Fig. 2:**
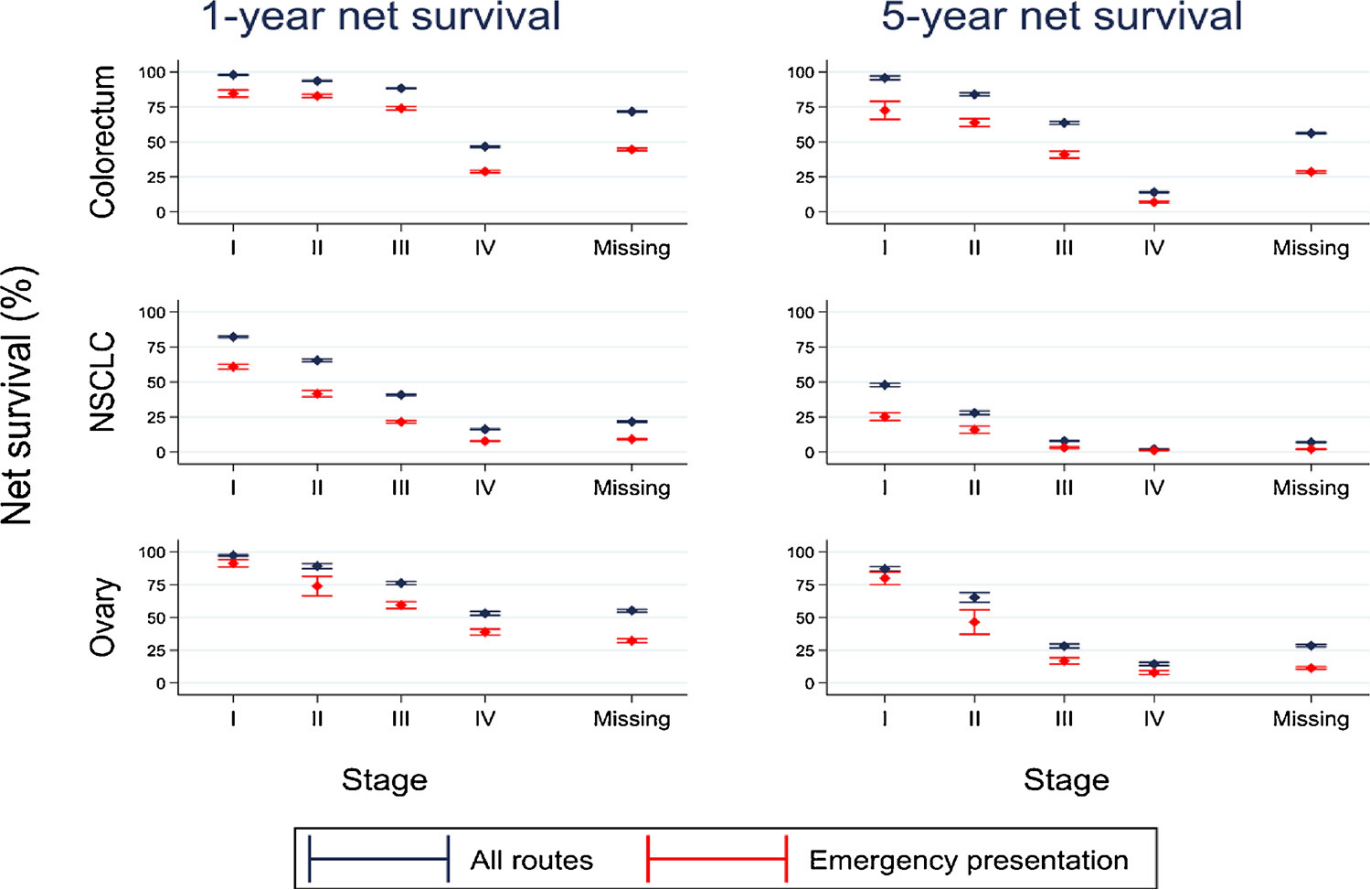
Net survival (with 95% confidence intervals (CI)) at one and five years by cancer, stage, and route to diagnosis, England.2009–2013.

One-year colorectal cancer survival was similar for patients diagnosed at stages I-III (9.6% difference between stages I and III) but markedly lower for stage IV patients (Table 3, Fig. 2). There was no such plateau in five-year survival (32.1% difference between stages I and III). For NSCLC and ovarian cancer, incremental differences in stage category (I vs II, II vs III, III vs IV) were associated with significantly lower one-year and five-year survival; no plateau was evident. Patients missing stage had low survival, typically between the survival of patients with stage III and stage IV disease (Table 3, Table 4).

**Table 3:** One-year net survival (and 95% confidence interval) by age group, route, cancer, and stage at diagnosis, England 2009–2013.

	Ages 15–59		Ages 60–79		Ages 80–99	
	All routes	Emergency presentation	All routes	Emergency presentation	All routes	Emergency presentation
Colorectal cancer						
I	99.3 (98.9, 99.7)	98.3 (96.7, 100.0)	98.8 (98.4, 99.1)	90.5 (87.2, 93.8)	93.7 (92.3, 95.1)	67.6 (61.9, 73.3)
II	97.8 (97.2, 98.3)	95.5 (93.9, 97.0)	95.2 (94.8, 95.6)	86.6 (85.1, 88.2)	87.7 (86.7, 88.8)	72.0 (69.5, 74.5)
III	95.4 (94.8, 96.0)	90.0 (88.0, 91.9)	90.6 (90.1, 91.1)	78.3 (76.7, 80.0)	76.2 (74.9, 77.5)	58.4 (55.7, 61.0)
IV	62.9 (61.7, 64.1)	48.9 (46.6, 51.2)	50.0 (49.3, 50.8)	31.2 (30.0, 32.5)	27.6 (26.6, 28.6)	15.2 (14.0, 16.5)
Missing	86.5 (85.7, 87.2)	75.6 (73.8, 77.5)	79.6 (79.1, 80.1)	52.1 (50.8, 53.4)	52.5 (51.7, 53.3)	30.2 (29.1, 31.3)
Total	85.6 (85.1, 86.0)	73.2 (72.0, 74.3)	80.7 (80.4, 81.0)	55.8 (55.0, 56.5)	59.9 (59.4, 60.4)	35.9 (35.0, 36.7)
Non-small cell lung cancer						
I	92.9 (91.8, 94.1)	84.9 (80.1, 89.8)	85.0 (84.3, 85.7)	67.0 (64.6, 69.4)	70.2 (68.6, 71.8)	49.2 (46.2, 52.2)
II	81.4 (79.1, 83.6)	70.7 (63.3, 78.0)	69.0 (67.8, 70.2)	45.9 (42.7, 49.0)	48.0 (45.9, 50.2)	30.5 (27.0, 34.0)
III	54.3 (52.8, 55.8)	34.6 (31.3, 37.9)	43.2 (42.5, 43.8)	23.5 (22.3, 24.8)	26.8 (25.8, 27.9)	14.6 (13.2, 16.0)
IV	24.0 (23.2, 24.9)	15.0 (13.9, 16.1)	16.9 (16.5, 17.2)	7.6 (7.3, 8.0)	10.0 (9.5, 10.5)	5.2 (4.8, 5.7)
Missing	39.4 (37.8, 41.1)	19.1 (17.0, 21.3)	24.4 (23.7, 25.0)	9.4 (8.8, 10.1)	12.3 (11.7, 12.9)	6.8 (6.2, 7.4)
Total	42.4 (41.7, 43.0)	22.1 (21.1, 23.1)	35.2 (34.9, 35.5)	14.7 (14.3, 15.1)	22.0 (21.6, 22.4)	10.7 (10.3, 11.2)
Ovarian cancer						
I	98.1 (97.4, 98.7)	96.0 (93.6, 98.4)	97.1 (96.1, 98.1)	90.3 (85.1, 95.5)	93.2 (89.2, 97.3)	64.4 (48.4, 80.4)
II	92.3 (89.7, 94.8)	80.4 (70.1, 90.8)	90.3 (87.6, 93.0)	83.7 (74.2, 93.2)	73.0 (64.3, 81.8)	36.4 (16.9, 56.0)
III	87.7 (86.0, 89.3)	79.7 (75.7, 83.8)	76.3 (74.8, 77.9)	59.7 (56.2, 63.1)	49.3 (45.3, 53.3)	27.2 (21.4, 33.0)
IV	72.2 (69.3, 75.1)	64.9 (59.9, 69.9)	57.3 (55.3, 59.4)	43.6 (40.4, 46.8)	21.4 (18.5, 24.2)	11.2 (8.3, 14.1)
Missing	82.0 (80.4, 83.5)	65.0 (61.3, 68.7)	58.4 (56.9, 59.8)	36.6 (34.2, 38.9)	22.7 (21.0, 24.5)	11.9 (10.1, 13.6)
Total	86.9 (86.1, 87.6)	73.9 (71.8, 76.0)	69.5 (68.7, 70.4)	46.8 (45.1, 48.4)	33.1 (31.7, 34.6)	14.8 (13.2, 16.3)

**Table 4:** Five-year net survival (and 95% confidence interval) by age group, route, cancer, and stage at diagnosis, England 2009–2013.

	Ages 15–59		Ages 60–79		Ages 80–99	
	All routes	Emergency presentation	All routes	Emergency presentation	All routes	Emergency presentation
Colorectum						
I	94.5 (92.6, 96.4)	93.4 (87.2, 99.5)	97.1 (95.7, 98.4)	74.4 (65.8, 82.9)	91.8 (86.2, 97.4)	58.4 (44.9, 71.9)
II	87.3 (85.6, 89.1)	78.4 (73.7, 83.2)	86.2 (85.0, 87.4)	68.5 (65.3, 71.8)	77.2 (73.7, 80.6)	50.6 (44.1, 57.0)
III	71.3 (69.4, 73.1)	57.5 (52.7, 62.2)	66.9 (65.7, 68.1)	42.3 (39.3, 45.4)	47.9 (44.9, 50.9)	29.7 (24.1, 35.2)
IV	19.7 (18.4, 20.9)	12.9 (11.0, 14.8)	15.3 (14.6, 16.0)	7.2 (6.3, 8.1)	7.3 (6.4, 8.3)	3.8 (2.6, 5.0)
Missing	68.9 (67.9, 69.8)	55.4 (53.3, 57.5)	64.2 (63.6, 64.8)	33.5 (32.3, 34.7)	37.7 (36.7, 38.7)	17.6 (16.4, 18.8)
Overall	63.2 (62.6, 63.9)	47.4 (45.9, 48.8)	62.0 (61.6, 62.5)	33.0 (32.2, 33.8)	42.9 (42.1, 43.8)	20.6 (19.5, 21.6)
NSCLC						
I	67.7 (65.0, 70.5)	52.6 (44.4, 60.7)	50.8 (49.4, 52.3)	29.7 (26.2, 33.1)	29.8 (26.8, 32.8)	13.3 (8.4, 18.1)
II	46.3 (42.7, 50.0)	43.1 (33.4, 52.7)	29.9 (28.1, 31.6)	18.5 (15.1, 21.9)	12.9 (10.0, 15.8)	6.0 (1.7, 10.3)
III	13.1 (11.9, 14.3)	7.5 (5.5, 9.6)	8.3 (7.8, 8.8)	3.3 (2.6, 4.0)	3.5 (2.8, 4.3)	1.2 (0.4, 2.0)
IV	3.9 (3.4, 4.3)	2.6 (2.0, 3.3)	1.8 (1.6, 2.0)	0.8 (0.6, 1.0)	1.3 (1.0, 1.7)	1.1 (0.6, 1.5)
Missing	17.9 (16.8, 19.0)	6.8 (5.6, 8.0)	7.9 (7.5, 8.3)	2.2 (1.9, 2.5)	2.5 (2.1, 2.8)	0.8 (0.5, 1.0)
Overall	16.6 (16.0, 17.1)	6.9 (6.2, 7.5)	11.5 (11.3, 11.8)	3.5 (3.2, 3.7)	5.3 (4.9, 5.6)	1.7 (1.4, 2.1)
Ovarian cancer						
I	88.3 (86.4, 90.2)	86.8 (82.1, 91.5)	86.5 (83.8, 89.3)	72.9 (63.2, 82.5)	81.6 (70.0, 93.1)	53.4 (26.5, 80.3)
II	74.7 (70.0, 79.4)	55.9 (42.4, 69.3)	63.1 (57.5, 68.7)	49.7 (34.6, 64.9)	37.2 (23.3, 51.1)	15.8 (-0.2, 31.8)
III	41.5 (38.4, 44.5)	31.5 (25.8, 37.2)	24.9 (22.9, 26.9)	14.4 (11.4, 17.4)	11.4 (7.4, 15.5)	3.6 (0.5, 6.6)
IV	26.6 (23.4, 29.8)	18.4 (13.5, 23.3)	13.8 (12.1, 15.5)	8.2 (6.1, 10.2)	3.6 (1.7, 5.4)	0.0 (0.0, 0.1)
Missing	55.2 (53.4, 57.0)	33.5 (30.2, 36.7)	25.1 (23.9, 26.2)	9.5 (8.2, 10.8)	8.1 (6.9, 9.4)	3.3 (2.2, 4.4)
Overall	57.9 (56.7, 59.1)	38.9 (36.5, 41.3)	31.7 (30.8, 32.6)	13.1 (12.0, 14.3)	13.4 (12.0, 14.8)	3.8 (2.7, 4.8)

#### 3.2.2 Emergency presentation: association with 30-day mortality and net survival

Twenty-three percent of colorectal, 35.6% of NSCLC, and 30.7% of ovarian cancer patients were diagnosed following emergency presentation (Appendix C in Supplementary data). Emergency presentation risk was greater for older patients diagnosed with NSCLC and ovarian cancer, whilst for colorectal cancer it was most common for patients aged 15–59 and 80–99 (Table 2).

Emergency presentation was associated with 1.9–2.9 times higher 30-day mortality (Table 2) and lower one-year net survival (Table 3): 50.7% for colorectal cancer compared with 75.9% survival for all routes combined; 14.1% compared to 32.6% for NSCLC; and 43.7% compared to 68.2% for ovarian cancer. Differences were greater for older patients. Similar patterns were observed for five-year survival (Table 4).

A small proportion (1.8–2.4%) of patients could not be assigned a route to diagnosis. Applying the assumption that these were all non-emergency presentations resulted in small changes in net survival (typically <1% and never >2%) indicating that these results are not sensitive to missing data (Appendix D in Supplementary data).

#### 3.2.3 Stage at diagnosis and emergency presentation: joint association with 30-day mortality and net survival

Patients diagnosed following emergency presentation were more likely to be diagnosed at stages III or IV or have stage missing (Table 2). Emergency presentation was associated with higher 30-day mortality and lower one- and five-year net survival for patients at each stage (Table 2, Table 3, Table 4). Survival differences between emergency and non-emergency colorectal cancer patients increased after the first year of follow up (Appendix E.1 in Supplementary data). By contrast, survival for emergency and non-emergency NSCLC patients converged to a ‘floor’ by the fifth year of follow up (Appendix E.2 in Supplementary data). Patients diagnosed following emergency presentation with missing stage had extremely high mortality: 26.4–51.0% died within 30 days following diagnosis (Table 2).

## 4 Discussion

Stage at diagnosis, emergency admission or presentation, and interval from first symptoms to diagnosis are commonly used indicators of early diagnosis. However, in the literature only stage and emergency diagnosis have a straightforward relationship with patient survival.

Our data analysis showed that emergency presentation and stage are independently associated with higher 30-day mortality and lower survival from colorectal, NSCLC and ovarian cancer in England. Patients without a recorded stage in population-based datasets had extremely high 30-day mortality and lower five-year survival.

### 4.1 Association between stage and survival

One-year survival from colorectal and breast cancers plateaued at stages I-III and was markedly lower at stage IV, whereas NSCLC and ovarian cancer displayed no such plateau. Five-year survival did not plateau at any stages for any cancer: each incremental increase in stage was associated with substantially lower survival. Granular information on stage at diagnosis, as opposed binary groupings, is therefore useful for monitoring progress in efforts to raise medium-term survival, although certain binary stage groupings may produce statistics which are strongly associated with short-term survival.

### 4.2 Association between emergency presentation and short-term mortality and survival

We found that emergency presentation was associated with higher 30-day mortality and lower medium-term survival for patients at every age and stage disease, consistent with other studies [44,45]. This indicator is therefore a proxy for other factors which independently determine survival, and is a valuable complimentary prognostic indicator to stage. However, more work is needed to understand why it is independently associated with survival.

### 4.3 Association between interval from first symptoms to diagnosis and short-term mortality and survival

Shorter intervals from first symptoms to diagnosis were not consistently associated with improved survival in the literature we examined. Other reviews concur. Neal et al. found instances of contradiction between studies on a given cancer on whether reducing the diagnostic, referral, or treatment interval was associated with higher survival or reduced mortality [46]. Hamilton et al found there was no consensus between experts that expediting symptomatic diagnosis conferred a mortality benefit for many common cancers [43].

These inconsistent findings may be partly explained by confounding by tumour aggressiveness and stage. The ‘waiting times paradox’ of the shortest intervals being associated with poor survival [42,47] is also likely to be partially attributable to confounding by these tumour factors [48]: Stage and aggressiveness may determine both type of first presenting symptoms (in turn determining interval length) and patient survival. We found evidence suggesting this in the literature: type of first symptoms was associated with the length of interval for childhood CNS [17], lung cancer [40], and ovarian cancer [20].

### 4.4 Monitoring performance using early diagnosis indicators

Monitoring of early-stage diagnosis is England is currently conducted using the percentage of patients diagnosed at stage I or II. However, we have shown that binary groupings of stage lose information which is predictive of medium-term survival. Numerical average stage (1–4) might provide a simple alternative measure that is more strongly associated with medium-term survival. Within a modelling framework ordered logistic regression with 4-category stage could be used instead of logistic regression with a binary stage indicator.

Emergency presentation is associated with advanced stage, and higher mortality and lower survival for patients at each stage. Data on emergency presentation could therefore be combined with stage information to generate a more informative prognostic index. Patients newly diagnosed through a given route at a given stage could be assigned a score which is the average survival of patients previously diagnosed with the same combination of indicators. For example, if a patient were diagnosed via emergency presentation at stage II disease, and previous 1-year survival for patients with these attributes was 80%, then that patient would be assigned a score of 80. The average score of the patient population could be then be used for monitoring and comparisons.

Our results don’t support the use of ‘average diagnostic interval length’ statistics for benchmarking and performance management. This is because the very shortest intervals are associated with poorer survival (due to confounding), so short intervals are not necessarily indicative of success in early diagnosis. However, it would be worthwhile to monitor whether reductions in average diagnostic intervals in response to an intervention in an area are associated with changes in the stage distribution or survival, to evaluate the effectiveness of the intervention.

Further work is also needed to identify alternative statistics based on the interval or similar quantities which are useful for surveillance. Alternative measures could include statistics on ‘missed opportunities’ for prompt symptomatic diagnosis: Lyratzopoulos et al. have described how these can occur [49], and Renzi et al identified instances of these for colorectal cancer [50].

We found that anatomical site of origin was strongly associated with probability of early diagnosis, regardless of the indicator used. The distribution of cancers should therefore be accounted for in performance comparisons, either through standardisation a modelling approach, to reduce bias from case-mix differences.

### 4.5 Interpreting missing stage information

In the English datasets we examined 16–36% of patients had no recorded stage. Compared to patients with a recorded stage, these patients had very high risk of death shortly following diagnosis and lower medium-term survival.

There are likely to be two different reasons why patients do not have a recorded stage. For most patients missing stage, it may be for administrative (non-clinical) reasons. These patients would have a similar stage distribution and survival to those with recorded stage. For a minority, it may have not been recorded *because* the patient was acutely unwell or had very poor prognosis at the time of first presentation. These patients would have more advanced disease, and many would die shortly after diagnosis. This hypothesis, that the majority of patients missing stage have typical stage and survival, and a minority have more advanced disease and very poor survival, explains the heterogeneity in 30-day mortality and survival we observed. It is also consistent with results from the study by Barclay et al suggesting that the stage distribution of these patients is slightly skewed towards later stages [51].

Our findings suggest that patients missing stage should be included in surveillance to avoid bias. This could be done using multiple imputation for missing data [52], using a ‘missing stage and died shortly following diagnosis’ percentage, or by applying expected survival statistics or model-based scores.

### 4.6 Strengths and limitations

We conducted a comprehensive joint analysis of the association between stage and emergency presentation with survival using 355,502 patient records, and compared results from this to the published literature. We also analysed the survival of patients missing a recorded stage, who comprise a substantial proportion of patients.

We restricted the literature search to documents explicitly mentioning ‘early diagnosis’. This approach gave us insight into what people consider ‘early diagnosis’ to encompass, however, it excluded studies where explicit mention of ‘early diagnosis’ was absent, so some data on the association between an indicator and patient survival may have been omitted.

### 4.7 Conclusion

In this study we identified the different indicators used to measure early diagnosis, and examined the association of each of the indicators with short-term mortality and survival. We recommend several changes to early diagnosis surveillance in England based on our findings: that granular stage information should be used in stage statistics to improve their prognostic value; that patients without a recorded stage should be included in surveillance to minimise bias; and that data on patient’s stage and route to diagnosis could be combined to create a composite early diagnosis indicator.

Shorter diagnostic intervals can be a result of late-stage disease and of patients being acutely unwell, and therefore we conclude that the average length of diagnostic interval is not an informative measure for performance management. More work is needed to examine the association between reductions in diagnostic interval length and survival improvements in an area, and to develop informative statistics based on the diagnostic interval for use in surveillance.

## Authorship contribution

PM and LW contributed to the study design. PM conducted the literature review, data analysis, and wrote a first draft of the manuscript under the guidance of LW. All authors contributed to interpretation of the study results and to editing the manuscript. All authors approved the final draft for submission.

## Conflicts of interest

None.
