# Supplementary material for: Which indicators of early cancer diagnosis from population-based data sources are associated with short-term mortality and survival?
Source: Cancer Epidemiol. 2018 Oct;56:161–70. doi: 10.1016/j.canep.2018.07.010 (PMC6189520; doi:10.1016/j.canep.2018.07.010)

**Supplementary Appendix**

**Appendix A: Literature search keywords and document inclusion criteria**

| **Elements*** | **PubMed search terms†** | **Google search terms‡** |
| --- | --- | --- |
| cancer | cancer* OR tumor* OR tumour* OR malignan* OR neoplasm* | cancer OR tumor OR tumour OR malignancy OR malignancies OR neoplasm |
| early diagnosis | "early cancer diagnosis" OR "earlier cancer diagnosis" OR "early diagnosis" OR "earlier diagnosis" | early cancer diagnosis OR earlier cancer diagnosis OR early diagnosis OR earlier diagnosis |
| population-based | "population-based" OR "population based" OR routine data* OR "routinely collected" | population-based OR population based OR routine data OR routinely collected |
| **Document inclusion criteria** | | |
| Criterion (1) | Includes presentation of new statistics or methods for generating statistics based on an explicitly defined indicator of early diagnosis from data sources that are or could be collected routinely | |
| Criterion (2) | Includes results describing the association between an early diagnosis indicator and patient prognosis (any measure of complications, mortality, survival, life expectancy, or cure). | |
| * "AND" operator was applied to only return documents containing all three elements   1. Title/abstract search 2. Different syntax was required to account for the differences in search options and logic implemented between PubMed and Google searches | | |

**Appendix B: Data Analysis Methods**

We linked together individual patient-level information from the different NHS datasets to create a summary stage variable for each patient, using the algorithm developed by Benitez-Majano *et al* [1]. The algorithm ensures all available stage information is used and prioritises information from clinical audits in case of conflicts. Standard criteria were used to decide whether a tumour record was eligible for inclusion in the analysis [2], and records were excluded if they contained data of inadequate quality or were for patients not resident in England. Patients known to have died on the same day as they were diagnosed (zero follow-up time) were included in the analyses. Patients for whom a death certificate was the only information available were excluded, because their duration of survival is unknown.

The period approach [3] was used to predict the five-year net survival [4] of patients who were diagnosed during 2009-2013, in the absence of complete follow-up data to five years for these patients. The period approach is analogous to the estimation of life expectancy at birth in a given year, where the most recently available mortality rates for each single year of age up to 99 years are used to estimate life expectancy. We predicted five-year survival for patients diagnosed in 2009-2013 (inclusive) by using follow-up data during the period 2009-2013 for patients diagnosed during 2006-2013, and applying the observed conditional probabilities of survival of these patients in each year of follow up to 5 years following diagnosis.

We used the Pohar Perme estimator of net survival [4], which estimates ‘survival from cancer’ in the absence of competing hazards. The excess hazard associated with cancer is estimated by comparing the observed hazard for cancer patients with the expected hazard for people with their demographic characteristics (age, sex, deprivation, etc) in population-based lifetables (also known as actuarial tables) [5]. We estimated net survival using the *stns* [6] command in the statistical software STATA 15 and UK deprivation-specific lifetables [7] (available for free at <http://csg.lshtm.ac.uk/tools-analysis/uk-life-tables/>). The exact STATA syntax used to estimate survival was:

stns list using LT_no_plateau_fixed, survival age(agediagindays=ageoutindays) ///

period(diagmdy=yearinday) ///

begintime(origin) strata(sex dep gor) rate(rate) citype(plain) ///

type(Ka) at(1 5, scalefactor(365.25) unit(year)) end_followup(end_fu)

This article describes in detail the implementation of the STNS command:

<https://www.stata-journal.com/sjpdf.html?articlenum=st0326>

**Data analysis methods references**

[1] S. Benitez-Majano, H. Fowler, C. Maringe, C. Di Girolamo, B. Rachet, Deriving stage at diagnosis from multiple population-based sources: colorectal and lung cancer in England, British journal of cancer 115(3) (2016) 391-400.

[2] R. Li, L. Abela, J. Moore, L.M. Woods, U. Nur, B. Rachet, C. Allemani, M.P. Coleman, Control of data quality for population-based cancer survival analysis, Cancer epidemiology 38(3) (2014) 314-20.

[3] H. Brenner, O. Gefeller, An alternative approach to monitoring cancer patient survival, Cancer 78(9) (1996) 2004-10.

[4] M.P. Perme, J. Stare, J. Estève, On Estimation in Relative Survival, Biometrics 68(1) (2012) 113-120.

[5] M.P. Perme, R. Henderson, J. Stare, An approach to estimation in relative survival regression, Biostatistics (Oxford, England) 10(1) (2009) 136-46.

[6] I. Clerc-Urmès, M. Grzebyk, G. Hédelin, Net survival estimation with stns, Stata Journal 14 (2014) 87-102.

[7] Department of the Environment Transport and the Regions, Measuring multiple deprivation at the small area level: the indices of deprivation 2000, London, 2000.

**Appendix C: Number of patients (%) diagnosed with colorectal, non-small cell lung, or ovarian cancer by route to diagnosis, England 2009-2013**

| Route to diagnosis | Colorectal cancer | Non-small cell lung cancer | Ovarian cancer |
| --- | --- | --- | --- |
| Death Certificate Only (DCO)  Emergency presentation  GP referral  Inpatient elective* Other outpatient†  Screening  Two-week wait (TWW)  Unknown‡ Missing | 70 (0.0)  37,285 (23.2)  38,253 (23.8)  5,800 (3.6) 11,488 (7.2)  15,046 (9.4)  45,898 (28.6) 3,939 (2.5)  2,838 (1.8) | 163 (0.1)  60,632 (35.6)  35,357 (20.7)  2,762 (1.6)  18,338 (10.8)  -  45,809 (26.9)  3,352 (2.0)  4,022 (2.4) | 15 (0.1)  7,503 (30.7)  5,291 (21.6)  282 (1.2)  2,597 (10.6)  -  7,485 (30.6)  734 (3.0)  543 (2.2) |
| Total | 160,617 (100.0) | 170,435 (100.0) | 24,450 (100.0) |
| * A route to diagnosis commencing with a planned inpatient admission   1. An elective route starting with an outpatient appointment 2. No records of inpatient, outpatient, or screening activity prior to diagnosis | | | |

**Appendix D: Arithmetic difference between five-year net survival calculated using a 'complete case' approach and a 'missing route is other route' approach for handling missing route to diagnosis data, by age and stage at diagnosis, England 2009-2013**

|  | All ages | Ages 15-59 | Ages 60-79 | Ages 80-99 |
| --- | --- | --- | --- | --- |
| Colorectal cancer | | | | |
| I | -0.3 | 0.1 | -0.2 | -1.0 |
| II | -0.4 | 0.0 | -0.4 | -0.6 |
| III | -0.4 | -0.1 | -0.3 | -0.6 |
| IV | -0.2 | -0.1 | -0.2 | -0.1 |
| Missing | -0.4 | -0.4 | -0.4 | -0.4 |
| Total | -0.5 | -0.2 | -0.4 | -0.6 |
| Non-small cell lung cancer | |  |  |  |
| I | -1.1 | -0.6 | -0.9 | -1.3 |
| II | -0.5 | -0.7 | -0.5 | -0.4 |
| III | -0.1 | -0.1 | -0.1 | 0.0 |
| IV | 0.0 | 0.0 | 0.0 | 0.1 |
| Missing | 0.2 | 0.0 | 0.2 | 0.1 |
| Total | -0.1 | -0.1 | 0.0 | -0.1 |
| Ovarian cancer |  |  |  |  |
| I | -0.1 | 0.0 | -0.1 | -0.7 |
| II | 0.0 | 0.3 | 0.0 | -1.2 |
| III | -0.2 | 0.0 | -0.2 | -0.1 |
| IV | 0.2 | 0.2 | -0.1 | 0.6 |
| Missing | 0.0 | 0.1 | -0.1 | 0.2 |
| Total | -0.1 | 0.1 | -0.2 | -0.1 |

**Appendix E.1: Colorectal cancer net survival by stage and route to diagnosis (all ages)**


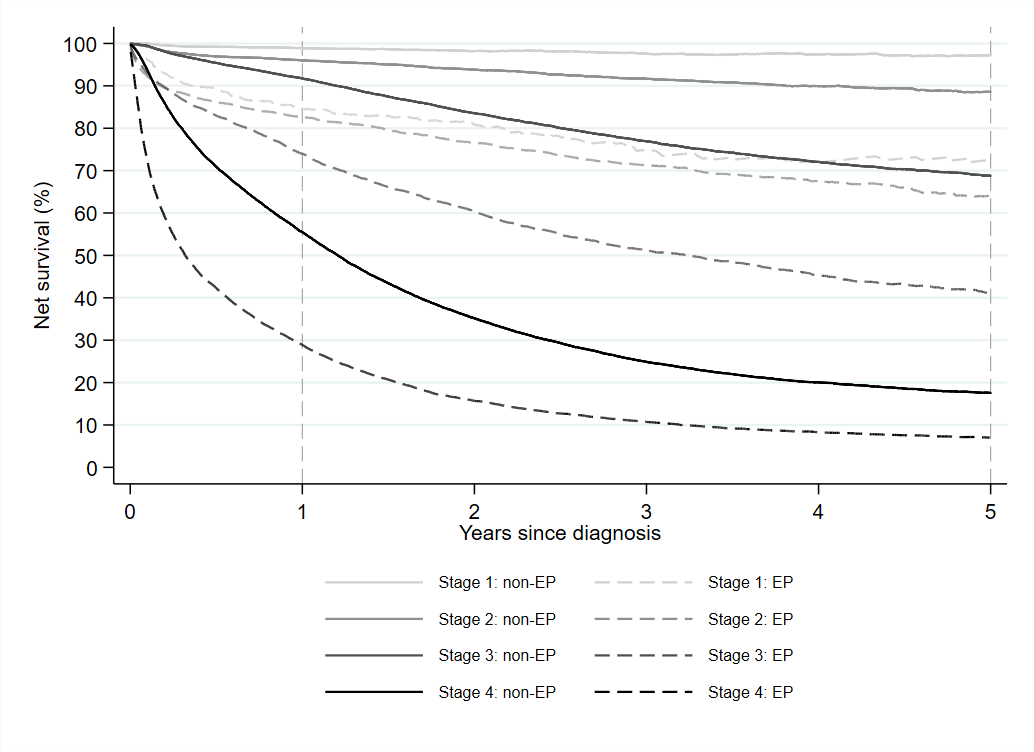


**Appendix E.2: NSCLC net survival by stage and route to diagnosis (all ages)**

**
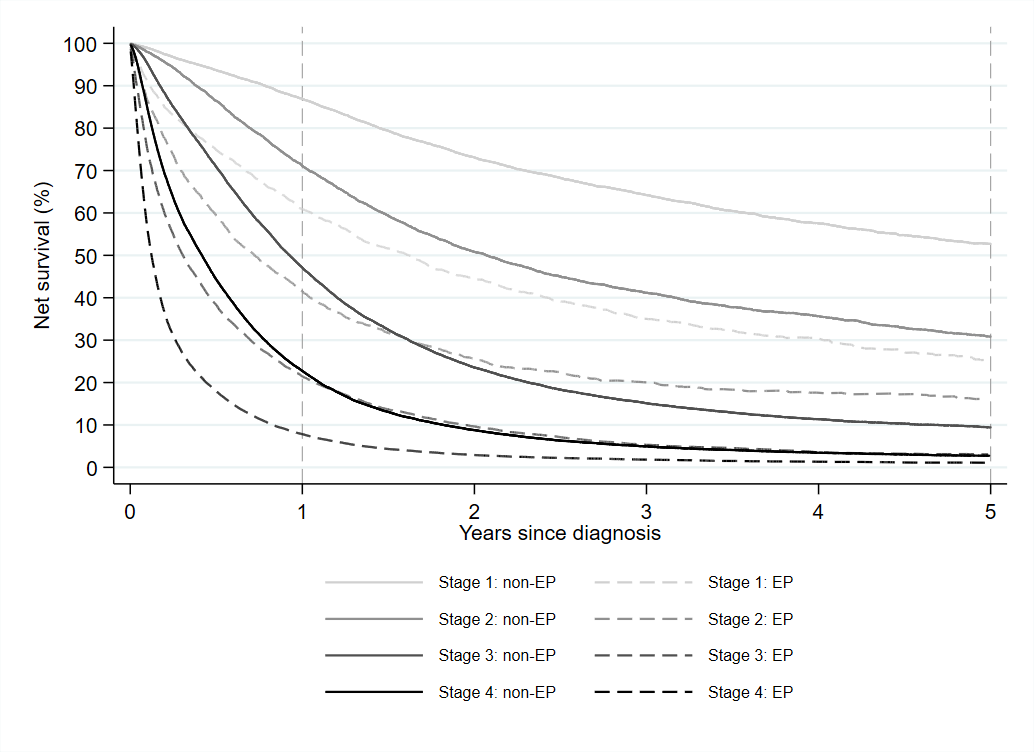
**

**Appendix E.3: Ovarian cancer net survival by stage and route to diagnosis (all ages)**


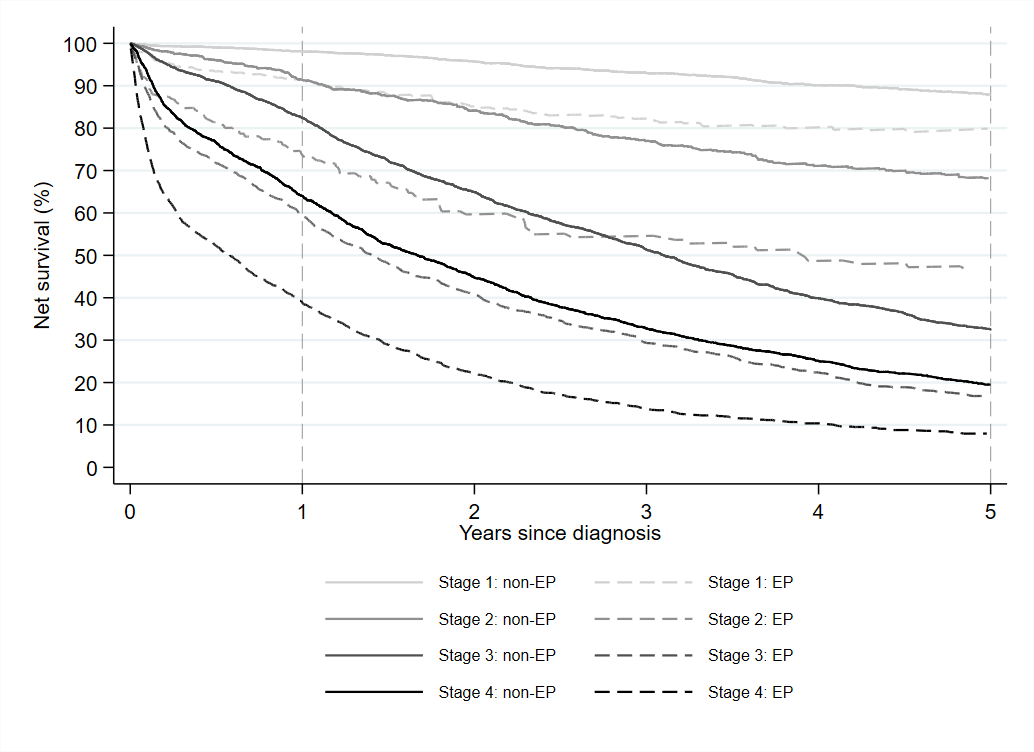

Supplement: Supplementary file 1 [file mmc1.docx]
